# Supplementary material for: Multi-site bone marrow core biopsy improves diagnostic accuracy in dogs with hematologic disease
Source: PLoS One. 2026 Feb 25;21(2):e0343251. doi: 10.1371/journal.pone.0343251 (PMC12935199; doi:10.1371/journal.pone.0343251)
Supplement: Attachment — Submitted filename: BMT in dogs.docx [file pone.0343251.s003.docx]

Thank you for the opportunity to review the manuscript entitled "Multi-Site Bone Marrow Core Biopsy Improves Diagnostic Accuracy in Dogs with Hematologic Disease".

This research aimed to evaluate diagnostic yield of single compared to multiple bone marrow biopsy sites in dogs. They found that increasing from one to two biopsy sites produced the largest increase in diagnostic yield and that quality of samples was better from iliac sites than humeral sites. There were site specific differences in myeloid-to-erythroid ratio and megakaryocyte counts and iron stores were higher in the ilium. There was fair agreement on the diagnoses on each biopsy core between the two pathologists that reviewed the marrows. There was generally fair to moderate agreement on the diagnoses for each pathologist across different anatomical sites of biopsy.

There are some concerns about the methodology and definitions of the study, which require attention.

Major:

- The authors acknowledged in the introduction that bone marrow aspirate and biopsy is required for bone marrow evaluation. However, it appears as if only bone marrow biopsy was performed in this study. Is that correct? And why was an aspirate not performed? Certain pathologies (e.g. MDS) can be diagnosed with greater accuracy if combined aspirate and trephine are evaluated.
- Iron grading is typically performed on bone marrow aspirate and graded according to Gale’s scale from 0-6. What iron grading system was used to grade iron on histology – I see it was grade 1-4, but there isn’t a reference to this grading system. Either it should be referenced or each grade explained. Were any dogs iron deficient? Was there agreement between sites and pathologists for an iron deficiency diagnosis? Or could that diagnosis not be made because there was no code for it in the code list?
- The diagnostic code list lists is a mixture of BM findings (hyperplasia, hypoplasia, BM inflammation, BM fibrosis) and real diagnoses (MDS, leukaemia, BM toxicity, metastaic neoplasia). BM findings are not diagnoses and rather point to a diagnosis that must still be made, e.g. BM hyperplasia may be reactive to infection, haemolysis or nutritional deficiency or may be clonal due to MDS or myeloproliferative neoplasm. Similarly, BM toxicity may present as either BM inflammation or BM hypoplasia depending on the toxin; both of these findings also on the code list. How was BM hyperplasia, BM hypoplasia, BM inflammation and BM toxicity defined? Were acute leukaemia and MDS defined according to WHO-HAEM5? Were each of these codes defined for the pathologists reviewing the marrows? I wonder whether the fact that there are both BM findings and BM diagnoses on the code list can explain the low (fair) agreement between pathologists, however there isn’t enough data provided currently to the reader (see next point).

There is also no diagnostic code for BM infection, despite one of the indications for BM examination being suspected BM infection.

- The descriptive data of the population is not included in the publication making it somewhat difficult to understand the findings. I suggest the following table.

| Dog | Path | Diagnosis | | | | | Iron grade | | | | | Quality | | | |
| --- | --- | --- | --- | --- | --- | --- | --- | --- | --- | --- | --- | --- | --- | --- | --- |
|  |  | LI | RI | LH | RH | Truth set | LI | RI | LH | RH | Average | LI | RI | LH | RH |
| 1 | 1 | 1 | 1 | 1 | 1 | 1,5 | 3 | 3 | 4 | 3 | 3 | Good | Good | Poor | Poor |
|  | 2 | 5 | 5 | 5 | 5 |  | 2 | 3 | 4 | 4 |  | Good | Good | Poor | Poor |
| 2 | 1 |  |  |  |  |  |  |  |  |  |  |  |  |  |  |
|  | 2 |  |  |  |  |  |  |  |  |  |  |  |  |  |  |
| 3 etc. | 1 |  |  |  |  |  |  |  |  |  |  |  |  |  |  |
|  | 2 |  |  |  |  |  |  |  |  |  |  |  |  |  |  |
| Proportion of poor/very poor quality | | | | | | | | | | | | 0% | | 100% | |

- The authors acknowledged that creation of the truth set had the limitation of being created by the most frequently used diagnostic codes for a dog (across all 4 sites and 2 pathologists). However, it would be more important to be able to pick up a real diagnosis (like a lymphoma), even if only at one site. Could the truth set be weighted so that a true diagnosis is included in the truth set (over a BM finding).
- The authors allude to the understanding that certain pathologies have patchy infiltration in the marrow. However, it is less clear whether the authors understand that this only pertains to certain disease entities. For example, aplastic anaemia, acute leukaemia, myeloproliferative neoplasms and myelodysplastic syndrome should be present in all of the marrow sites as by nature they are stem cell diseases, and for these disease entities a single biopsy is generally acceptable. However infiltrating diseases such as myeloma, lymphoma, granuloma and metastatic solid tumours have patchy infiltration requiring multiple biopsies. Even with that being said, the multiple biopsies are not necessarily required from multiple anatomical sites, as long as >20mm of marrow is collected. <https://pubmed.ncbi.nlm.nih.gov/12562655/>. It must come across in the introduction that multiple biopsy sites is one of the methods used to improve diagnostic yield (but not the only).

On this point, the diagnostic codes for leukaemia and round cell neoplasia were combined into one, despite having different marrow infiltration patterns. Would it be possible to separate those entities and report them separately? It would be interesting to see whether acute leukaemia and MDS were indeed found across all biopsy sites, as expected.

Minor:

- Do the clinical pathologists have experience with looking at bone marrow biopsies and do they have experience with veterinary/canine bone marrows?
- How was quality defined? Length of biopsy? Number of intertrabecular spaces?

It is stated that quality was nondiagnostic, poor, adequate, good or exclellent. But then in the results speaks about ordinal scale – please state how the two are related.

- How was non-diagnostic defined? Did this mean that the marrow was of very poor quality or did it mean that the marrow was normocellular with no pathology? If the former, were there no normal marrows in the sample? Or no code for normal marrows so that was not an option?
- It is also not clear if nondiagnostic codes overlapped on the same biopsies between pathologists – hence the need for the suggested table above.
- Define what is considered round cell neoplasia within the methods.
- What is the cut-off of lymphocytosis and palsmacytosis of 5% and 2% based on? (in a normal human marrow the normal range for BM lymphocytes is 6-20%). How was this evaluated – purely morphologically? Or was immunohistochemistry used to highlight the lymphocytes and plasma cells?
- Do reference ranges exist for MER, cellularity and megakaryocyte numbers in dogs? It is expected to find differences between dogs in this study because these dogs had different pathologies (as opposed to healthy dogs, where a difference between may reflect true inter-breed variability and need for breed specific reference ranges).
- In the subscript of Table 1 where the nondiagnostic distribution is described, why is the denominator by site 18 if there were 16 dogs?
- Table 4: What is a *sign p*? It is not clear why three different p values are provided.
- Table 10: In the subscript, the statement *(ICCs above are: 53%, 65%, 23%, and 12% respectively)* seems unnecessary as it is in the table.
- *“Pairwise site comparisons confirmed that logMER was significantly reduced at LH (p=0.0096), LI (p=0.0412), and RH (p=0.0082) when contrasted with RI.”* Were RI and LI similar and were RH and LH similar? While we can expect differences between ilium and humerus, we do not expect differences between right and left. Biologically that would not make sense and if found, then there may be a confounder that needs to be explained.

Same can be said for megakaryocyte counts (unless there is displacement of haematopoiesis by an infiltrate at one biopsy site). Currently the statement is “*Sampling site significantly affected the mean megakaryocyte numbers (p=0.006), with LI samples containing significantly fewer megakaryocytes than other sites.”* But what would be more meaningful to evaluate is whether LI and RI were similar, LH and RH were similar, and perhaps iliac sites were different from humeral sites, as this is what is expected from literature.

- Table 11: What is Num DF and Den DF? Add to abbreviation list under the table.
- Would it be appropriate to conclude that, since iliac crest samples have better quality and better iron stores and that the largest improvement in diagnostic yield occurs from 1 to 2 biopsies, that it is recommended 9based on these study results) that bilateral iliac crest biopsies is advised.

It is stated in the discussion that “*the complementary information obtained from*

*humeral sites suggests that sampling both flat and long bones may be optimal*” – what is that complementary information that is helpful?

- *This study provides robust evidence supporting multi-site bone marrow sampling as a superior diagnostic approach compared to current single-site standards in veterinary medicine.* – This study has proven that multi-site biopsy is better than a single biopsy from a single site. It has not proven that multi-site biopsy is better than multiple biopsies from the same anatomical site.
