# Supplementary material for: Multi-site bone marrow core biopsy improves diagnostic accuracy in dogs with hematologic disease
Source: PLoS One. 2026 Feb 25;21(2):e0343251. doi: 10.1371/journal.pone.0343251 (PMC12935199; doi:10.1371/journal.pone.0343251)
Supplement: Attachment — Submitted filename: 25.docx [file pone.0343251.s004.docx]

**PlosOne Review	NR	8 October 2025**

**Multi-Site Bone Marrow Core Biopsy Improves Diagnostic Accuracy in Dogs with**

**Hematologic Disease**

1. “limitations of single-site sampling have been particularly evident in human hematologic oncology, where multi-region sampling approaches have shown superior diagnostic yields compared to conventional single-site methods.[23]” gives the impression that multi-site sampling is the norm in humans. “However, despite these advances in human medicine, veterinary hematology has largely maintained single-site sampling protocols” Again, mentioned in the Discussion “The observed benefit aligns with findings from human oncology, where multi-site tumor ampling has been shown to outperform routine single-site approaches in detecting intratumoral heterogeneity and high-grade disease components. [20, 21, 23]” Whilst this data is correct, these few studies have not impacted routine practice in humans. This thread of canine medicine lagging behind humans for BMAT sampling is inaccurate and requires correction. Marrow infiltration in humans has also evolved to incorporate imaging (PET-CT). Whilst not a focus in this paper, has this been considered in dogs?
2. The reasoning for the randomization of the site of BM aspiration in the methodology needs to be clearly explained.
3. Where there any failed marrow aspirate and trephine samples? Did the non-diagnostic reads include failed samples?
4. “However, the substantial inter-dog variability observed for iron stores (ICC=0.72) suggests that patient-specific variables, including the underlying disease state of each dog, also significantly influence sample characteristics. This variability emphasizes that sample quality is determined by both anatomical location and patient-specific factors, highlighting why multi-site sampling provides a crucial buffer against sampling inadequacy from any single location.[29, 38, 39]” This paragraph in the Discussion requires re-wording. It is expected that inter-dog and underlying disease will affect findings (including iron stores). So, the link between patient factors and multi-site sampling is unclear. Much of this paragraph is unnecessary.
5. The secondary objectives were to determine optimal number and sample site combinations. Whilst the site sample number is clear addressed in the Discussion, the site combination discussion is more vague. Was the study able to determine an optimal site combination?
6. “These findings align with emerging understanding of bone marrow spatial heterogeneity and suggest that multi-site sampling should be considered as a new standard of care for veterinary bone marrow evaluation” The sample size is too small to justify this conclusion; would suggest re-wording.
