# Supplementary material for: Multi-site bone marrow core biopsy improves diagnostic accuracy in dogs with hematologic disease
Source: PLoS One. 2026 Feb 25;21(2):e0343251. doi: 10.1371/journal.pone.0343251 (PMC12935199; doi:10.1371/journal.pone.0343251)
Supplement: Attachment — Submitted filename: Response to Reviewers.docx [file pone.0343251.s006.docx]

**The authors thank the Reviewers and Editor for the time and effort put into providing critique to improve our manuscript.**

Reviewer #1: Thank you for the opportunity to review the manuscript entitled "Multi-Site Bone Marrow Core Biopsy Improves Diagnostic Accuracy in Dogs with Hematologic Disease".

AUTHORS’ RESPONSE: We appreciate your thoroughness and rigor in critically evaluating our manuscript, and we have implemented many of your suggestions. Notably, we really appreciated the idea to add the Table that captures the Truth Set, the Truth Rules, and the Sample Quality and Iron. In doing so, we discovered that our initial secondary-aims model inadvertently included nondiagnostic reads; we have now re-run those analyses excluding nondiagnostic cores, to match the primary analysis set, which changed the absolute values but not the direction of the results from our secondary-aims models.

This research aimed to evaluate diagnostic yield of single compared to multiple bone marrow biopsy sites in dogs. They found that increasing from one to two biopsy sites produced the largest increase in diagnostic yield and that quality of samples was better from iliac sites than humeral sites. There were site specific differences in myeloid-to-erythroid ratio and megakaryocyte counts and iron stores were higher in the ilium. There was fair agreement on the diagnoses on each biopsy core between the two pathologists that reviewed the marrows. There was generally fair to moderate agreement on the diagnoses for each pathologist across different anatomical sites of biopsy. There are some concerns about the methodology and definitions of the study, which require attention. Please see the attached document for details.

Major:

The authors acknowledged in the introduction that bone marrow aspirate and biopsy is required for bone marrow evaluation. However, it appears as if only bone marrow biopsy was performed in this study. Is that correct? And why was an aspirate not performed? Certain pathologies (e.g. MDS) can be diagnosed with greater accuracy if combined aspirate and trephine are evaluated.

AUTHORS’ RESPONSE: Thank you for this important comment. You are correct that the present manuscript focuses exclusively on bone marrow core biopsies. Bone marrow aspirates were also collected from each dog during the same procedure, but the cytologic findings and their correlation with biopsy results are presented in a separate manuscript currently under review in another journal. Combining both datasets in a single paper would have substantially exceeded the scope and length limits of this study. We considered the biopsy component independently appropriate for this analysis, as trephine sections represent a distinct and complementary diagnostic modality that allows evaluation of marrow architecture, fibrosis, and infiltrative disease, parameters that cannot be reliably assessed cytologically. The companion manuscript addresses the complementary cytologic data, ensuring that each modality is examined in adequate depth.

Iron grading is typically performed on bone marrow aspirate and graded according to Gale’s scale from 0-6. What iron grading system was used to grade iron on histology – I see it was grade 1-4, but there isn’t a reference to this grading system. Either it should be referenced or each grade explained.

AUTHORS’ RESPONSE: Thank you for this thoughtful comment. In veterinary medicine, the assessment of iron stores in cytologic marrow samples is far less defined than in human medicine. The Gale method has been evaluated in low numbers of animals in two recent studies (2021, doi: 10.1111/vcp.12947; 2023, doi: 10.1111/vcp.13209). Due to the small physical size of many patients, it is relatively common that marrow particles of insufficient number and/or size are obtained for application of the Gale method. Additionally, Prussian Blue staining of marrow samples is not automatically performed on veterinary bone marrow cytology specimens. Consequently, evaluation and scoring of iron stores on cytology remain subjective and ill-defined, without consistent acceptance and application of a given method.

To our knowledge, the Gale system has not been validated for histologic assessment of core biopsy sections in veterinary species, including dogs. Iron distribution and staining characteristics differ substantially between aspirate smears and decalcified trephine sections. Indeed, a J Clin Pathol study (2005; DOI: 10.1136/jcp.2004.017038) comparing iron staining in aspirates and decalcified trephine biopsies from 155 human bone marrow specimens demonstrated that aspirate smears reflect marrow iron stores more reliably than decalcified sections, likely due to iron loss during decalcification. In that study, a 0–4 grading scale was applied for histologic sections. Similarly, we employed a 4-point ordinal rubric as a study-specific adaptation to enhance reproducibility and facilitate direct site-to-site comparisons within dogs. Each grade (1–4) corresponded to increasing iron deposition across 10 random 40× microscopic fields, and the operational definitions for these categories have now been provided in the Methods for clarity:

“…1-4 ordinal iron stores score (1=absent; 2=minimal [rare, small foci of faintly visible granules]; 3=moderate [clearly visible multifocal deposits in macrophages or along trabeculae]; 4=abundant [dense, coalescing granules widely distributed throughout the marrow]; based on 10 random 40× microscopic fields)…”

Were any dogs iron-deficient? Was there agreement between sites and pathologists for an iron deficiency diagnosis? Or could that diagnosis not be made because there was no code for it in the code list?

AUTHORS’ RESPONSE: Thank you for this comment. The present study was not designed to assess specific disease entities such as iron deficiency but rather to evaluate the diagnostic capture probability and site-specific variability of bone marrow core biopsy interpretation in dogs with suspected hematologic disease. As outlined in our stated objectives, the focus was on assessing how the number and anatomical distribution of trephine biopsy sites influence diagnostic accuracy, sample quality, and inter-pathologist agreement.

The diagnosis of iron deficiency typically requires integration of multiple parameters, including Prussian blue–stained **bone marrow aspirates** (doi: 10.1136/jcp.2004.017038; doi:10.1111/j.1751-553X.2008.01100.x), serum iron indices, and erythrocyte morphology. In contrast, iron evaluation on decalcified trephine core sections is limited by both technical factors (iron loss during decalcification) and biologic variability (spatial heterogeneity of iron deposition across marrow regions). As a result, biopsy-based iron grading alone cannot distinguish true systemic iron deficiency from physiologic variability in local iron storage. Therefore, a specific diagnostic code for iron deficiency was not included in the code list (Table 2a), as this condition was outside the intended scope of our study.

The diagnostic code list lists is a mixture of BM findings (hyperplasia, hypoplasia, BM inflammation, BM fibrosis) and real diagnoses (MDS, leukaemia, BM toxicity, metastaic neoplasia). BM findings are not diagnoses and rather point to a diagnosis that must still be made, e.g. BM hyperplasia may be reactive to infection, haemolysis or nutritional deficiency or may be clonal due to MDS or myeloproliferative neoplasm. Similarly, BM toxicity may present as either BM inflammation or BM hypoplasia depending on the toxin; both of these findings also on the code list. How was BM hyperplasia, BM hypoplasia, BM inflammation and BM toxicity defined? Were acute leukaemia and MDS defined according to WHO-HAEM5? Were each of these codes defined for the pathologists reviewing the marrows? I wonder whether the fact that there are both BM findings and BM diagnoses on the code list can explain the low (fair) agreement between pathologists, however there isn’t enough data provided currently to the reader (see next point). There is also no diagnostic code for BM infection, despite one of the indications for BM examination being suspected BM infection.

AUTHORS’ RESPONSE: Thank you for this thoughtful comment. Our code list was intentionally designed as a morphology-driven classification appropriate for trephine core biopsy interpretation in dogs. In routine veterinary reporting, pathologists often record both pattern-level morphologic impressions (e.g., hyperplasia, hypoplasia, inflammation, fibrosis) and disease-level categories (e.g., leukemia/round-cell neoplasia, metastatic neoplasia). Because this study evaluated how the number and anatomical distribution of histologic core biopsies (with CBC) affect diagnostic capture, sample quality, and inter-pathologist agreement, rather than performing full disease work-ups, we adopted a concise code set that could reflect the spectrum of patterns seen on decalcified sections while capturing clear disease entities, avoiding excessive granularity that would underpower site-level comparisons. The two blinded board-certified pathologists were provided a written codebook (now added as Supplementary Table S2) with operational definitions and coding rules. They were instructed to prioritize disease-level codes when definitive features were present and use pattern-level codes when findings were morphologic but non-definitive. Leukemia/round-cell neoplasia and MDS were separated morphologically using blast burden and architectural/cytologic features on cores plus CBC; however, we did not apply full WHO-HAEM5 diagnostic criteria because immunophenotyping, cytogenetics, and molecular testing were beyond the scope of this sampling-strategy study and are mostly unavailable for use in dogs. We recognize that mixing pattern- and disease-level categories can modestly lower inter-observer agreement in morphology-only assessments, but our primary endpoint, diagnostic capture as a function of site number/combination, is robust to such granularity. A separate “BM infection” code was not included because definitive infection requires ancillary confirmation; suspected infectious cases on morphology were captured under “BM inflammation,” and Table 2a has been revised to state this explicitly. For transparency, Methods now reference Supplementary Table S2 (Morphologic Codebook) summarizing the definitions and rater instructions.

The descriptive data of the population is not included in the publication making it somewhat difficult to understand the findings. I suggest the following table.

| Dog | Path | Diagnosis | | | | | Iron grade | | | | | Quality | | | |
| --- | --- | --- | --- | --- | --- | --- | --- | --- | --- | --- | --- | --- | --- | --- | --- |
|  |  | LI | RI | LH | RH | Truth set | LI | RI | LH | RH | Average | LI | RI | LH | RH |
| 1 | 1 | 1 | 1 | 1 | 1 | 1,5 | 3 | 3 | 4 | 3 | 3 | Good | Good | Poor | Poor |
|  | 2 | 5 | 5 | 5 | 5 |  | 2 | 3 | 4 | 4 |  | Good | Good | Poor | Poor |
| 2 | 1 |  |  |  |  |  |  |  |  |  |  |  |  |  |  |
|  | 2 |  |  |  |  |  |  |  |  |  |  |  |  |  |  |
| 3 etc. | 1 |  |  |  |  |  |  |  |  |  |  |  |  |  |  |
|  | 2 |  |  |  |  |  |  |  |  |  |  |  |  |  |  |
| Proportion of poor/very poor quality | | | | | | | | | | | | 0% | | 100% | |

AUTHORS’ RESPONSE: We generated SUPP TABLE S1 per Reviewer request. This has been an invaluable idea.

The authors acknowledged that creation of the truth set had the limitation of being created by the most frequently used diagnostic codes for a dog (across all 4 sites and 2 pathologists). However, it would be more important to be able to pick up a real diagnosis (like a lymphoma), even if only at one site. Could the truth set be weighted so that a true diagnosis is included in the truth set (over a BM finding).

AUTHORS’ RESPONSE: Thank you for this thoughtful point. Our aim in defining the truth set was to avoid circularity in the absence of a gold standard. We therefore used an unsupervised, frequency-based dog-level consensus across all 8 reads (4 sites × 2 pathologists). This behaves like a pre-test probability: it selects the label(s) most supported by the totality of reads without assigning diagnosis-specific priors. Methodologically, this limits incorporation bias, reduces the influence of site-specific outliers (e.g., a single atypical field on one slide), and keeps performance estimates comparable across categories. By contrast, forcing “true diagnoses” (e.g., lymphoma) into the truth set whenever they appear at one site effectively introduces supervision through an informative prior. That approach increases sensitivity to focal disease but also risks false positives and spectrum/verification bias, especially when slide quality varies, and requires post-hoc choices about which codes qualify as “true.”

The authors allude to the understanding that certain pathologies have patchy infiltration in the marrow. However, it is less clear whether the authors understand that this only pertains to certain disease entities. For example, aplastic anaemia, acute leukaemia, myeloproliferative neoplasms and myelodysplastic syndrome should be present in all of the marrow sites as by nature they are stem cell diseases, and for these disease entities a single biopsy is generally acceptable. However infiltrating diseases such as myeloma, lymphoma, granuloma and metastatic solid tumours have patchy infiltration requiring multiple biopsies. Even with that being said, the multiple biopsies are not necessarily required from multiple anatomical sites, as long as >20mm of marrow is collected. <https://pubmed.ncbi.nlm.nih.gov/12562655/>. It must come across in the introduction that multiple biopsy sites is one of the methods used to improve diagnostic yield (but not the only).

AUTHORS’ RESPONSE: The relevant part in the introduction has been revised as follows to address the Reviewer’s comment: “The limitations of single-site sampling have been highlighted in human hematologic oncology, where a limited number of studies demonstrated that multi-region sampling can improve the detection of focal or spatially heterogeneous disease compared to conventional single-site methods.[23] These findings support the broader understanding that hematopoietic disorders may exhibit regionally distributed lesions rather than uniform involvement, suggesting that multi-site sampling could enhance diagnostic accuracy in selected human conditions.[24, 25] In veterinary hematology, however, single-site sampling protocols remain standard practice,[10] and the potential diagnostic benefit of multi-site approaches for canine bone marrow evaluation has not been systematically investigated.[12, 13, 26]”

On this point, the diagnostic codes for leukaemia and round cell neoplasia were combined into one, despite having different marrow infiltration patterns. Would it be possible to separate those entities and report them separately? It would be interesting to see whether acute leukaemia and MDS were indeed found across all biopsy sites, as expected.

AUTHORS’ RESPONSE: We appreciate the interest in site-distribution patterns. However, separating “leukemia” from “round-cell neoplasia” in our dataset is not methodologically defensible. This study deliberately limited interpretation to H&E morphology without immunophenotyping, flow cytometry, PARR testing, or cytogenetics. Under these conditions, canine marrow cannot be reliably subclassified into myeloid vs lymphoid lineages (or acute leukemia vs lymphoma/plasma-cell neoplasia) at the level the Reviewer requests. Implementing a post-hoc split would therefore create false precision, increase misclassification risk, and introduce incorporation/circularity bias by effectively re-labeling cases using assumptions we cannot verify with the routine histopathology.

Our codebook pre-specified a single category (Code 3) for “leukemia/round-cell tumor” precisely to preserve reproducibility across readers using routine stains. The aim of this work was to evaluate cross-site and cross-reader consistency under routine histomorphologic conditions, not to adjudicate disease-specific biology that requires orthogonal methods. Splitting Code 3 after seeing the data would be post-hoc and statistically underpowered, and would undermine comparability across sites and dogs.

For transparency, Supplementary Table S1 (the “Dog × Path × Site” matrix) already shows that Code 3 appears in both multi-site and site-limited patterns across dogs, for example, some dogs have Code 3 at multiple sites while another site in the same dog is non-3, illustrating that mixed topography occurs in practice on H&E and cannot be mapped cleanly to “diffuse vs patchy” entities without immunophenotyping. Inferring that any given Code-3 case is “acute leukemia/MDS” (diffuse) versus “lymphoma/myeloma” (patchy) is therefore speculative in this dataset.

Accordingly, we will retain the pre-specified combined Code 3 in the primary analyses. To meet the reviewer’s interest without over-interpreting, we will (i) add a brief results sentence summarizing the within-dog, across-site distribution of Code 3 calls (already visible in Supplementary Table S1), and (ii) note explicitly in the Limitations that resolving leukemia subtypes and distinguishing leukemia from other round-cell neoplasms would require IHC/flow/cytogenetics.

Minor:

Do the clinical pathologists have experience with looking at bone marrow biopsies and do they have experience with veterinary/canine bone marrows?

AUTHORS’ RESPONSE: Yes. In veterinary medicine, it is common for clinical pathologists to be trained in and perform histologic evaluation of bone marrow specimens. Both study readers are experienced veterinary clinical pathologists with substantial expertise in evaluation of canine bone-marrow histopathology and cytology. In particular, Dr. Raskin is widely regarded as a top expert in veterinary bone-marrow evaluation and has trained multiple cohorts of anatomic and clinical pathologists (including AG [anatomic pathologist] and SC [clinical pathologist]) in bone-marrow histopathologic assessment. At the University of Illinois, SC and AG routinely interpret bone-marrow histopathology in clinical practice (AG reads the internal medicine bone marrows).

To minimize bias, the study PI (AG, who is also a board-certified small-animal internist), who managed the enrolled cases, did not participate in the blinded reads or adjudication. The two readers evaluated the slides independently and were blinded, ensuring that diagnostic calls reflected their domain expertise rather than case management knowledge. This combination, experienced readers plus blinding, supports the reliability of the marrow interpretations used in the study.

How was quality defined? Length of biopsy? Number of intertrabecular spaces?

AUTHORS’ RESPONSE: We did not prespecify objective QC metrics (e.g., core length or a minimum number of evaluable inter-trabecular spaces). Instead, each core’s quality was judged subjectively by the blinded clinical pathologist rater using a 4-level, **practice-based** scale: 1 = nondiagnostic, 2 = poor, 3 = adequate, 4 = excellent. Judgments reflected overall evaluability (cellularity and preservation of architecture), degree of hemorrhage/dilution and crush artifact, decalcification/processing artifacts, cortical contamination, representation of inter-trabecular spaces, and stain quality. We did not count inter-trabecular spaces or measure core length systematically. We recognize the absence of hard QC thresholds as a limitation; however, **it also reflects current veterinary practice, where universally adopted quantitative marrow QC criteria are absent**.

For analyses, we collapsed this ordinal score into two categories to stabilize estimates and mirror real-world reporting: “Poor” = score 2 and “Good” = scores 3–4. “Nondiagnostic” (score 1) and “Missing” (missing data) were excluded from the “scorable” denominator in “Proportion of poor” analyses, as shown in the “All” vs “Scorable” summaries in Supp Table S1.

It is stated that quality was nondiagnostic, poor, adequate, good or exclellent. But then in the results speaks about ordinal scale – please state how the two are related.

AUTHORS’ RESPONSE: Quality was scored on a four-level ordered (ordinal) scale per core: 1 = non-diagnostic, 2 = poor, 3 = adequate, 4 = excellent. We refer to it as an ordinal scale because the categories have a natural order, but we do not assume equal spacing between levels. For presentation in the Supp Table S1 which the Reviewer requested, we collapsed levels 2 as “Poor” and 3–4 as “Good,” while reporting 1 (“non-diagnostic”) separately; missing values are shown as blank. All analyses that reference “ordinal quality” use this ordered 1–4 coding.

How was non-diagnostic defined? Did this mean that the marrow was of very poor quality or did it mean that the marrow was normocellular with no pathology? If the former, were there no normal marrows in the sample? Or no code for normal marrows so that was not an option?

AUTHORS’ RESPONSE: By “non-diagnostic” we mean technically inadequate material that cannot be interpreted, not “marrow appears normal.” In practice, the non-diagnostic label was assigned when the trephine core/sections lacked sufficient evaluable marrow or were obscured by artifacts that precluded assessment. These cases received the non-diagnostic code and a correspondingly lowest quality score, and, as prespecified, were excluded from truth-set and rule-based analyses to avoid conflating poor specimen quality with biology.

“Non-diagnostic” therefore does not mean “normocellular with no pathology.” A case with adequate tissue that truly lacked an abnormality would not be labeled non-diagnostic. In our clinical cohort, dogs were sampled for compelling clinical indications; consequently, we did not observe marrows judged “within normal limits,” and our code set did not include a separate “normal” category. Adding a hypothetical “normal” code would not change our central agreement/consistency endpoints, but retaining a non-diagnostic bucket was essential to prevent technical failures from biasing diagnostic performance.

It is also not clear if nondiagnostic codes overlapped on the same biopsies between pathologists – hence the need for the suggested table above.

AUTHORS’ RESPONSE: As stated previously, we made the requested table (SUPP TABLE S1).

Define what is considered round cell neoplasia within the methods.

AUTHORS’ RESPONSE: In our Methods, “round-cell neoplasia” refers to a neoplastic infiltrate of monomorphic round cells identified on H&E-stained sections from FFPE trephine cores, as determined by the pathologist’s histomorphologic assessment. Operationally, this label was applied when round cells expanded or replaced intertrabecular marrow and showed cytologic atypia with architectural effacement beyond what is expected for reactive conditions. This umbrella category encompasses lymphoid neoplasms (e.g., lymphoma, leukemic involvement), plasma-cell neoplasia (myeloma), mast-cell neoplasia, and undifferentiated round-cell malignancies when recognizable on H&E alone. Conversely, purely reactive/physiologic lymphoid aggregates without atypia or effacement were not coded as neoplasia. For analysis, these cases were mapped to our prespecified composite code “3 = leukemia/round-cell neoplasia.” As explained previously, the aims and scope of the study did not include differentiating different leukemic types from other round cell tumors.

We intentionally did not subdivide by lineage (e.g., myeloid vs lymphoid) because this dataset did not include immunophenotyping (IHC/flow) or cytogenetics; attempting to force lineage-specific calls on H&E alone would increase misclassification risk and bias the unsupervised, frequency-based consensus we used to construct dog-level truth labels. When H&E suggested lineage-tilt (e.g., plasmacytoid features), this was captured descriptively in the read, but the analytic code remained within the single “round-cell neoplasia” category to preserve specificity and comparability across sites/readers. This conservative definition is aligned with our study’s aim, site/pathologist agreement, and diagnostic yield, while explicitly acknowledging that definitive subtyping requires adjunct tests and is a priority for future prospective work.

What is the cut-off of lymphocytosis and palsmacytosis of 5% and 2% based on? (in a normal human marrow the normal range for BM lymphocytes is 6-20%). How was this evaluated – purely morphologically? Or was immunohistochemistry used to highlight the lymphocytes and plasma cells?

AUTHORS’ RESPONSE: Our thresholds (≥5% lymphocytes; ≥2% plasma cells) were pre-specified screening cut-offs for canine marrow, chosen to flag uncommon expansions rather than to establish a diagnosis (PMID: 22285155). Two points are key: 1) Species and method differences. Normal canine trephine cores on H&E typically contain few mature lymphocytes and rare plasma cells relative to human marrow, and histologic estimates on FFPE cores differ from cytologic differentials on aspirates. Using conservative, low cut-offs allowed us to identify conspicuous increases on routine H&E without over-calling normal background cellularity. 2) Consistency and avoidance of bias. The same cut-offs were applied uniformly across readers and dogs to avoid post-hoc tuning. Cells were assessed purely morphologically on H&E (with PAS as indicated); no IHC/flow was performed in this retrospective cohort. Lymphocytes were recognized by small size and clumped chromatin; plasma cells by eccentric nuclei, coarse “clock-face” chromatin, and a paranuclear clearing. Counts were semi-quantitative across multiple HPFs, and the binary flags (above/below the prespecified cut-off) were used only as secondary descriptors, not as primary diagnostic endpoints.

Do reference ranges exist for MER, cellularity and megakaryocyte numbers in dogs? It is expected to find differences between dogs in this study because these dogs had different pathologies (as opposed to healthy dogs, where a difference between may reflect true inter-breed variability and need for breed specific reference ranges).

AUTHORS’ RESPONSE: Breed-specific reference intervals for marrow indices are not established in dogs. Standard veterinary texts describe normal cellularity as broadly ~25–75%, and myeloid-to-erythroid ratio (MER) in healthy marrow as ~0.9–1.8; notably, MER derived from histology can trend higher than cytology because of sectioning and fixation artifacts (we previously reported higher histologic MER in healthy dogs: Gal et al., Vet Pathol 2018;55:853–860). For megakaryocytes, Schalm’s Textbook of Veterinary Hematology emphasizes that enumeration is inherently semi-quantitative due to irregular spatial distribution; typical means reported are approximately 2.76 per low-power field (drop-squash) and 1.55 per low-power field (particle-squash), and routine assessment relies on scanning multiple spicule-containing fields/trabecular spaces rather than applying a narrow “reference range.” In other words, robust, breed-specific reference intervals for megakaryocyte number are not available, and the literature cautions against over-precision in these counts.

Given our study population comprised sick dogs with diverse diseases, between-dog differences in cellularity, MER, and megakaryocyte counts are expected and clinically appropriate. The question we specifically interrogated is within-dog, across-site consistency: in truly diffuse processes, site-to-site measures should be similar, whereas patchy or regionally heterogeneous disease (or sampling variability) will produce site effects. Our results (Table 10) demonstrate significant site-level differences in MER, megakaryocytes, and blasts within dogs, supporting the biological and sampling rationale for multi-site assessment. Thus, while healthy-dog reference intervals provide useful context, our design and findings address a different, and clinically salient issue: how marrow indices vary by site within the same patient, which directly informs procedural strategy and diagnostic yield.

In the subscript of Table 1 where the nondiagnostic distribution is described, why is the denominator by site 18 if there were 16 dogs?

AUTHORS’ RESPONSE: Thanks for flagging the ambiguity. In Table 1, the nondiagnostic denominator is site-level, not dog-level. Each dog can contribute up to four anatomical sites (LI, RI, LH, RH), and more than one site in the same dog can be nondiagnostic. Across the 16 dogs, there were 18 nondiagnostic biopsy sites; hence the site denominator is 18 even though there are 16 dogs. We have revised the table legend to make this explicit.

Table 4: What is a *sign p*? It is not clear why three different p values are provided.

AUTHORS’ RESPONSE: In Table 4, “Sign p” is the p-value from the exact binomial sign test applied to the within-dog paired differences (Δp) for each adjacent contrast; it tests whether improvements and declines are equally likely (H₀: P[Δp>0]=0.5), excluding ties. We reported three p-values because they address different aspects and assumptions of Δp’s distribution: the paired t-test evaluates the mean difference under approximate normality; the Wilcoxon signed-rank test evaluates the median difference using ranks (direction and magnitude) with weaker distributional assumptions; and the sign test is fully distribution-free and uses direction only, making it robust to outliers and unequal magnitudes. To prevent confusion, we clarified in the table subscript that the Wilcoxon test was prespecified as the primary analysis.

Table 10: In the subscript, the statement *(ICCs above are: 53%, 65%, 23%, and 12% respectively)* seems unnecessary as it is in the table.

AUTHORS’ RESPONSE: We removed the repetition in the table subscript.

*“Pairwise site comparisons confirmed that logMER was significantly reduced at LH (p=0.0096), LI (p=0.0412), and RH (p=0.0082) when contrasted with RI.”* Were RI and LI similar and were RH and LH similar? While we can expect differences between ilium and humerus, we do not expect differences between right and left. Biologically that would not make sense and if found, then there may be a confounder that needs to be explained.

AUTHORS’ RESPONSE: As we explained in the beginning, we discovered that our initial secondary-aims model inadvertently included nondiagnostic reads; we have now re-run those analyses excluding nondiagnostic cores, to match the primary analysis set. In the updated mixed-effects model (fixed effects: site and pathologist; random intercept: dog; Tukey adjustment for pairwise tests), least-squares mean logMER values are lower at the humeral sites than at the right ilium: LH vs RI p=0.0309 and RH vs RI p=0.0092. The left ilium vs right ilium is not significant (LI vs RI p=0.1375), and left vs right comparisons are not significant for either bone (LH vs RH and LI vs RI both non-significant after Tukey correction). Thus, the updated results align with biological expectations, no lateral (left–right) asymmetry, while showing a bone-level effect (humerus vs ilium) that is anatomically plausible.

Same can be said for megakaryocyte counts (unless there is displacement of haematopoiesis by an infiltrate at one biopsy site). Currently the statement is “*Sampling site significantly affected the mean megakaryocyte numbers (p=0.006), with LI samples containing significantly fewer megakaryocytes than other sites.”* But what would be more meaningful to evaluate is whether LI and RI were similar, LH and RH were similar, and perhaps iliac sites were different from humeral sites, as this is what is expected from literature.

AUTHORS’ RESPONSE: As stated above, when we reran the analysis excluding nondiagnostic samples, there is no lateral (left–right) asymmetry. The revised section reads as follows: “Sampling site significantly affected the mean megakaryocyte numbers (p=0.0026), with LI samples containing significantly fewer megakaryocytes than LH (p=0.0084) and RH (p=0.0034); RI did not significantly differ from the other sites.”

Table 11: What is Num DF and Den DF? Add to abbreviation list under the table.

AUTHORS’ RESPONSE: In the subscript under the table we added “Num DF = numerator degrees of freedom for the test; Den DF = denominator degrees of freedom”.

Would it be appropriate to conclude that, since iliac crest samples have better quality and better iron stores and that the largest improvement in diagnostic yield occurs from 1 to 2 biopsies, that it is recommended 9based on these study results) that bilateral iliac crest biopsies is advised.

AUTHORS’ RESPONSE: Not exactly. Our data support using two sites, but they also show site-specific strengths that argue against taking both cores from the same anatomic region. Iliac crest biopsies yielded higher sample quality and more robust iron stores, whereas humeral sites showed slightly higher rule-based diagnostic correctness. Because the largest jump in yield occurs when moving from one to two biopsies, the most efficient way to capitalize on that gain is to sample complementary niches, one flat bone (ilium) and one long bone (humerus), rather than two iliac crests. Bilateral iliac sampling would duplicate correlated information and risks missing patchy processes preferentially involving long bones, while an ilium+humerus strategy leverages the ilium’s quality/iron advantages and the humerus’s correctness advantage. Accordingly, based on these results, we recommend one iliac crest plus one humeral biopsy when obtaining two cores, rather than bilateral iliac crests.

It is stated in the discussion that “*the complementary information obtained from*

*The humeral sites suggest that sampling both flat and long bones may be optimal.*” – What is that complementary information that is helpful?

AUTHORS’ RESPONSE: By “complementary information,” we mean that the humeral cores contributed non-redundant diagnostic value along several, orthogonal axes that the iliac crest cores did not consistently provide on their own: 1) Diagnostic correctness signal (rule-based performance): In our dog-level, rule-based framework (ANY / BOTH_any / BOTH_same against the unsupervised truth set), humeral sites frequently yielded a correct code when the paired iliac site did not, and vice versa. This within-dog, across-site discordance indicates that errors are not perfectly correlated across flat and long bones; adding a humeral site therefore measurably lowers the probability of a joint miss (the largest gain in capture probability occurs when moving from one to two sites in Table 4), which is the practical definition of complementary diagnostic information. 2.) Different strengths in ancillary histologic features: Site effects were observed in metrics related to sample quality and iron grading, whereas the categorical diagnosis signal (correctness) showed a somewhat different site pattern. In other words, the long-bone and flat-bone cores do not contribute the same thing: one site is more likely to give you an interpretable, iron-informative core in a given dog, while the other may be more likely to align with the dog-level truth set. Using both sites increases the chance that the final case record contains both (a) a scorable/representative core for ancillary interpretation and (b) a code that matches the dog’s consensus diagnosis. 3) Biologic and sampling heterogeneity: Flat (ilium) and long (humerus) bones differ in trabecular architecture, adiposity, vascular microanatomy, and the spatial distribution of hematopoietic elements. Diseases with patchy infiltration are plausibly enriched or more readily appreciable in one niche over the other. This biologic non-equivalence translates into site-specific detectability, so sampling both niches captures heterogeneity that a bilateral single-region strategy may miss. Taken together, these points explain our statement: humeral cores add complementary value because they (i) reduce correlated error with iliac cores in the rule-based diagnostic analysis, (ii) fill gaps when iliac cores are less informative for quality/iron (or the reverse), and (iii) sample a different marrow niche where patchy disease may be detectable. That is why, when two cores are obtained, our data support pairing one iliac crest with one humeral site rather than duplicating a single anatomic region.

*This study provides robust evidence supporting multi-site bone marrow sampling as a superior diagnostic approach compared to current single-site standards in veterinary medicine.* – This study has proven that multi-site biopsy is better than a single biopsy from a single site. It has not proven that multi-site biopsy is better than multiple biopsies from the same anatomical site.

AUTHORS’ RESPONSE: We appreciate the distinction. Our study was designed to test whether sampling more than one anatomic site improves diagnostic capture relative to a single-site standard; it was not powered to compare “two cores from one site” versus “one core from each of two sites.” That said, our data and biologic rationale support prioritizing two different sites when a second core is taken. First, within-dog analyses showed meaningful across-site discordance in correctness (and differences in ancillary features such as iron and scorable quality), indicating that iliac crest and humerus contribute non-redundant information. This lowers the joint miss probability more than duplicating a single anatomic region would be expected to do, because errors are less correlated across different marrow niches (flat vs long bone) than within the same niche. Second, many target diseases in our cohort are patchy, and site effects we observed imply site-specific detectability; sampling two distinct compartments therefore better addresses spatial heterogeneity than repeating the same site. We agree our study does not directly randomize “two cores same site” versus “two different sites,” so we have revised the wording to be precise: “our results demonstrate that moving from one to two sites materially increases the probability of capturing a correct diagnosis, and support choosing two distinct anatomic sites to maximize complementary yield.” We now state this explicitly in the Discussion and note in the Limitations that a head-to-head comparison of “two cores same site” vs “two sites” is an important next step.

**Reviewer #2**: 1.“Limitations of single-site sampling have been particularly evident in human hematologic oncology, where multi-region sampling approaches have shown superior diagnostic yields compared to conventional single-site methods.[23]” gives the impression that multi-site sampling is the norm in humans. “However, despite these advances in human medicine, veterinary hematology has largely maintained single-site sampling protocols” Again, mentioned in the Discussion “The observed benefit aligns with findings from human oncology, where multi-site tumor ampling has been shown to outperform routine single-site approaches in detecting intratumoral heterogeneity and high-grade disease components. [20, 21, 23]” Whilst this data is correct, these few studies have not impacted routine practice in humans. This thread of canine medicine lagging behind humans for BMAT sampling is inaccurate and requires correction.

AUTHORS’ RESPONSE: Thank you for this thoughtful comment. We appreciate the clarification and have revised the paragraph to avoid overstating the clinical adoption of multi-site sampling in human hematology while retaining the conceptual parallels relevant to our study. The revised text now reads as follows:

“The limitations of single-site sampling have been highlighted in human hematologic oncology, where a limited number of studies demonstrated that multi-region sampling can improve the detection of focal or spatially heterogeneous disease compared to conventional single-site methods.[23] These findings support the broader understanding that hematopoietic disorders may exhibit regionally distributed lesions rather than uniform involvement, suggesting that multi-site sampling could enhance diagnostic accuracy in selected human conditions.[24, 25] In veterinary hematology, however, single-site sampling protocols remain standard practice [10], and the potential diagnostic benefit of multi-site approaches for canine bone marrow evaluation has not been systematically investigated.[12, 13, 26]”

Marrow infiltration in humans has also evolved to incorporate imaging (PET-CT). Whilst not a focus in this paper, has this been considered in dogs?

AUTHORS’ RESPONSE: Thank you for this comment. We agree that advanced imaging modalities such as PET-CT have increasingly been integrated into human hematologic oncology to assess marrow infiltration and spatial disease burden. In veterinary medicine, however, PET-CT remains largely confined to research settings due to limited accessibility, high cost, and the lack of validated protocols for hematologic applications in dogs. As such, it has not yet become part of routine diagnostic practice, and our study focused on histopathologic sampling rather than imaging-based assessment.

2.The reasoning for the randomization of the site of BM aspiration in the methodology needs to be clearly explained.

AUTHORS’ RESPONSE: Thank you for this comment. The randomization of bone marrow sampling order was implemented to eliminate potential sequence effects, such as changes in operator technique, fatigue, or minor procedural variability, that could systematically bias sample quality or diagnostic yield at specific sites. By randomizing the order of site sampling across dogs, we ensured that any such effects were evenly distributed and did not confound site-based comparisons. This is now explained in the relevant method section.

3. Were there any failed marrow aspirate and trephine samples? Did the non-diagnostic reads include failed samples?

AUTHORS’ RESPONSE: Thank you for this question. As indicated in Table 2b, two bone marrow core biopsies could not be obtained and were recorded as missing data. The nondiagnostic category, by contrast, included cores that were successfully acquired but deemed uninterpretable due to inadequate marrow content, crush artifact, or other quality-related limitations. Thus, nondiagnostic reads reflect samples of insufficient diagnostic quality rather than failed collection attempts.

4.“However, the substantial inter-dog variability observed for iron stores (ICC=0.72) suggests that patient-specific variables, including the underlying disease state of each dog, also significantly influence sample characteristics. This variability emphasizes that sample quality is determined by both anatomical location and patient-specific factors, highlighting why multi-site sampling provides a crucial buffer against sampling inadequacy from any single location.[29, 38, 39]” This paragraph in the Discussion requires re-wording. It is expected that inter-dog and underlying disease will affect findings (including iron stores). So, the link between patient factors and multi-site sampling is unclear. Much of this paragraph is unnecessary.

AUTHORS’ RESPONSE: Thank you for this helpful comment. We agree that inter-dog variability and disease-related differences in marrow characteristics are expected and that our previous wording overstated their connection to the rationale for multi-site sampling. We have revised the paragraph to focus on the key message while removing unnecessary interpretation, as follows:

“Substantial inter-dog variability in iron stores (ICC = 0.70) was observed, reflecting biological diversity and differences related to disease status among dogs. These findings indicate that, beyond anatomical site effects, individual patient factors also influence marrow composition. Nonetheless, the significant site-dependent differences identified in our study demonstrate that sampling location itself contributes independently to diagnostic variability, supporting the value of multi-site evaluation.[29, 38, 39]”

5.The secondary objectives were to determine optimal number and sample site combinations. Whilst the site sample number is clear addressed in the Discussion, the site combination discussion is more vague. Was the study able to determine an optimal site combination?

AUTHORS’ RESPONSE: Thank you for this insightful comment. The study was not specifically powered to test all possible site combinations statistically; however, the site-specific results (Tables 5 and 12) allow reasonable inference about optimal pairings. Iliac crest samples consistently provided higher sample quality and iron stores, whereas humeral sites, particularly the right humerus, showed slightly higher per-read correctness. Taken together, these data suggest that combining one iliac and one humeral site yields complementary diagnostic information and represents the most informative and practical two-site combination. We have clarified this interpretation in the revised Discussion.

6.“These findings align with emerging understanding of bone marrow spatial heterogeneity and suggest that multi-site sampling should be considered as a new standard of care for veterinary bone marrow evaluation” The sample size is too small to justify this conclusion; would suggest re-wording.

AUTHORS’ RESPONSE: Thank you for this comment. We agree that the sample size (n=16 dogs) is relatively modest; however, several aspects of the study design strengthen confidence in the observed effect. The study was prospectively conducted, randomized, and blinded, with multiple independent site evaluations per dog (64 biopsies, 128 reads), yielding a large number of within-subject comparisons. The improvement in diagnostic capture from one to two sites was highly significant (P < 0.0001) with non-overlapping confidence intervals, making a type I error unlikely. We therefore maintain that the direction and magnitude of this effect are robust, although we have tempered the language in the Discussion to avoid implying that multi-site sampling is already an established “standard of care.” The revised sentence now reads as follows:

“These findings align with emerging understanding of bone-marrow spatial heterogeneity and support consideration of multi-site sampling as a best-practice approach to improve diagnostic accuracy in veterinary bone-marrow evaluation.”
