# Supplementary material for: Multi-site bone marrow core biopsy improves diagnostic accuracy in dogs with hematologic disease
Source: PLoS One. 2026 Feb 25;21(2):e0343251. doi: 10.1371/journal.pone.0343251 (PMC12935199; doi:10.1371/journal.pone.0343251)
Supplement: Attachment — Submitted filename: Response_to_Reviewers_auresp_2.docx [file pone.0343251.s007.docx]

**The authors thank the Reviewer and Editor for the time and effort put into providing critique to improve our manuscript.**

Reviewer #1: Thank you for the excellent explanations in the response to the reviewers. The following information was explained in the response to the reviewer, but is not in the methods and would interest the reader or enhance clarity:
- The pathologists were experienced veterinary clinical pathologists
- Immunohistochemistry was not performed
AUTHORS’ RESPONSE: The information the Reviewer mentioned above is now included in the text.

Given the new information provided with the supplementary tables, I have a couple of queries:
Supplementary Table S1:
- In Table S1, there are the columns ANY sites, BOTH_any sites and BOTH_same sites. Do these correlate to the rules ANY, BOTH_any and BOTH_same? If not, then the columns should be labelled differently as they are currently labelled too similar to the rules. I assumed they were referring to the rules for the next comments.

AUTHORS’ RESPONSE: We changed the column heading to match the rules (i.e., Any, Both_any, and Both_same)

- From the table, it is evident that BOTH_any and BOTH_same was only different for one dog (dog 12), where the truth set had 2 codes. For all other dogs, the truth set had one code and then BOTH_any was the same as BOTH_same. This brings into question the definitions of BOTH_any and BOTH_same.
- Definition of BOTH_any in Table S1 appears to indicate both pathologists were correct at the same site (otherwise dog 13 would have been included as both pathologist had the correct codes, but since the codes were correct at different sites, it was not included). This does not correspond to the BOTH_any definition supplied in the methods (both correct, not necessarily the same code), or in Table 3 (both in truth set). The same definition should be used consistently across different sections/tables and be easy to follow in the dataset.
- Similar applies to BOTH_same (correct with the same code at the same sites, as it appears in Table S1 vs ‘both agree on truth set’ vs ‘both agree and correct’ vs ‘both pathologists agree on a correct diagnosis’). If the site didn’t matter as implied by the definition ‘both agree on the truth set’, then dog 13 should have been included.

AUTHORS’ RESPONSE:

We appreciate the reviewer’s careful reading. The key clarification is that the rules **ANY**, **BOTH_any**, and **BOTH_same** are evaluated **within a given site** (LI, RI, LH, RH). In other words, a site is counted under a rule only based on the two pathologists’ interpretations **at that same site**; agreement (or correctness) occurring at different sites does not qualify.

**First, a scenario where the “truth set” has ties:**

Site:   LI  LH  RI  RH

PATH1:  A  B  A  C

PATH2:  B  C  B  A

TRUTH SET: {A, B} because there are 3 × A and 3 × B

- **RULE ANY:** LI, LH, RI, RH (each site has at least one call in {A,B})
- **RULE BOTH_any:** LI, RI (at these sites, **both pathologists’ diagnoses fall within the truth set {A,B}**, even though they selected different codes).
- **RULE BOTH_same:** none (there is **no site where both pathologists selected the same truth-set code**).

This example illustrates why **BOTH_any** and **BOTH_same** can differ when the truth set has ties: within a site, the two pathologists can each be “in the truth set” while choosing different tied codes.

**Second, a scenario where the “truth set” has no ties:**

Site:   LI  LH  RI  RH

PATH1:  A  B  A  C

PATH2:  B  B  B  A

TRUTH SET: {B} because there are 4 × B

- **RULE ANY:** LI, LH, RI (sites with at least one B)
- **RULE BOTH_any:** LH (only LH has BB)
- **RULE BOTH_same:** LH (only LH has BB)

This example shows that when the truth set is a **single code**, **BOTH_any** and **BOTH_same** are necessarily identical, because “both pathologists within the truth set” implies both selected the same single truth-set code.

Therefore, **BOTH_any and BOTH_same differ only when** (1) the truth set contains ties, and (2) within the same site one pathologist selects one truth-set code while the other selects the other truth-set code.

Regarding **Dog 13**, the truth set contains a single code (no ties), and there is **no site** at which both pathologists selected the truth-set code. Although each pathologist selected the truth-set code at some site, they did not do so at the **same site**, so **BOTH_any** and **BOTH_same** are correctly empty for Dog 13 under the within-site definitions.

To ensure consistent terminology across the manuscript, we revised the Methods and the Table 3 footnote to explicitly reflect the within-site nature of these rules:

- **ANY:** within a site, at least one pathologist is within the truth set.
- **BOTH_any:** within a site, both pathologists are within the truth set (when the truth set has ties, they may not necessarily choose the same truth-set code).
- **BOTH_same:** within a site, both pathologists are within the truth set **and** both choose the same truth-set code (even when there are ties).

According to these definitions, Supplementary Table S1 is correct. We also reordered the BM sampling sites in Supplementary Table S1 to match the order used when listing sites under the “Truth set” (LH, LI, RH, RI) to improve readability.

Supplementary Table S2:
- For MDS: Can the mentioned dysplastic features be seen on trephine biopsy? Many of those features would typically be evident on bone marrow aspirate and not histology.

AUTHORS’ RESPONSE: Thank you for this comment. We agree that several dysplastic features commonly used to support a diagnosis of myelodysplastic syndrome (MDS), particularly subtle erythroid and granulocytic abnormalities, are generally more readily appreciated on bone marrow aspirate cytology than on trephine histology. Supplementary Table S2 lists diagnostic criteria for MDS rather than features that are uniquely or optimally assessed on trephine sections alone.

In the context of trephine evaluation, marrow cellularity and blast proportion (including blast aggregation/clustering) can be assessed on histologic sections, and megakaryocytic abnormalities are often appreciable. While recognition of erythroid and granulocytic dysplasia on histology is more challenging, these features can be identified in trephine sections by experienced hematopathologists, albeit with less sensitivity than on aspirate smears. Accordingly, such features were interpreted cautiously on histology and considered supportive rather than definitive in isolation.

Thus, Supplementary Table S2 reflects standard diagnostic criteria for MDS, whereas the relative ease of identifying individual features depends on the specimen type and was accounted for during interpretation.

Figure 2 and Figure 3:
- The legend is currently unhelpful. The line is presumably the 95% CI and the circle the mean. It should be labelled accordingly.

AUTHORS’ RESPONSE: legends have been revised as follows:

**Figure 2.** Model-based site effects on per-reading correctness. GLIMMIX least-squares means with 95% CIs for LH, LI, RH, RI on the probability scale. No site effect (p=0.599) and no pathologist effect (p=0.781). **Point:** LS-mean predicted probability; **Horizontal line:** 95% CI.

**Figure 3.** Inter-pathologist agreement by site pair. Simple κ with 95% CIs for each site pair pooled across pathologists, with N overlaid. Highlights that LI–RH shows the highest agreement. **Open circle:** κ estimate; **Horizontal line:** 95% CI.

- As the Figures 2 and 3 contain the same information as Table 3 and Table 5, I suggest omitting the tables.

AUTHORS’ RESPONSE:

We thank the Reviewer for this suggestion. While we agree that Tables 3 and 5 and Figures 2 and 3 summarize overlapping results, we respectfully believe that the tables and figures provide complementary, rather than redundant, presentations of the data, and that retaining both enhances clarity for readers with different preferences for data interpretation.

Specifically, the tables provide precise numerical estimates and confidence intervals that allow detailed comparison across conditions, whereas the figures convey the same results in a visual format that facilitates rapid assessment of effect size, uncertainty, and overlap across sites and site pairs. In our experience, these two formats support distinct cognitive approaches to understanding the results, quantitative scrutiny versus pattern recognition, and are commonly used together for this reason.

Because the study addresses spatial heterogeneity and agreement across biopsy sites, we believe the graphical displays are particularly valuable for visually communicating site-level trends and uncertainty that are less immediately apparent from tabular data alone, while the tables remain essential for exact reporting.

For these reasons, we respectfully request that the Reviewer reconsider the suggestion to omit either the tables or the figures. We would also welcome the Editor’s guidance on whether retaining both formats is appropriate for the journal and readership.

Table 6 adds limited information. I suggest omitting it and including the 95% CI in the text.

Authors' Response: Thank you for this suggestion. We have omitted Table 6 and incorporated the key results, including the 95% confidence intervals, into the Results section. The remaining tables have been renumbered consecutively.
